# Supplementary material for: Alternative Tissue Sampling for Improved Detection of Candidatus Liberibacter asiaticus
Source: Plants (Basel). 2023 Sep 23;12(19):3364. doi: 10.3390/plants12193364 (PMC10574540; doi:10.3390/plants12193364)
Supplement: Supplementary file 1 [file plants-12-03364-s001.zip › plants-2603764-supplementary.pdf]

Table S1: Types of trees, or exposure risk categories, tested in the study.

Description	# Trees	# Trees with Positive Tissue	# Sampling Events	# Sampling Events Positive
Known-Positive[a]	336	303	337	304
ACP+ site[b]	27	11	152	15
Inconclusive (ACP)[c]	4	2	24	4
Inconclusive (Plant)[d]	27	10	176	12
K9 Alert[e]	10	2	56	3
K9 Survey[f]	2	1	10	1
Misc. Category	2	0	10	0
Total	408	329	765	339

[a] Known-Positive: Tree confirmed *C*Las-positive by CDFA during a regular survey at a previous time point.

[b] ACP+ site: ACP sample from a residential property tested positive for *C*Las.

[c] Inconclusive (ACP): ACP sample from a residential property tested inconclusive for *C*Las.

[d] Inconclusive (Plant): Plant sample from a residential property tested inconclusive for *C*Las.

[e] K9 Alert: Residential tree alerted by HLB detection dog (F1 K9).

[f] K9 Survey: Trees part of K9 survey but not alerted by HLB detection dog.

Table S2. RNR/COX primers and probes sequences [11].

Primert/ProbeName	Sequences 5′-3′
RNR-F primer	CAT GCT CCA TGA AGC TAC CC
RNR-R primer	GGA GCA TTT AAC CCC ACG AA
RNR-probe	5-FAM/CCT CGA AAT CGC CTA TGC AC/3BHQ-1
COX-F primer	GTA TGC CAC GTC GCA TTC CAG A
COX-R primer	GCC AAA ACT GCT AAG GGC ATT C
COX-probe	5-VIC/ATC CAG ATG CTT ACG CTG G/3BHQ-2
